# Evolutionary rates and patterns for human transcription factor binding sites derived from repetitive DNA

**DOI:** 10.1186/1471-2164-9-226

**Published:** 2008-05-17

**Authors:** Nalini Polavarapu, Leonardo Mariño-Ramírez, David Landsman, John F McDonald, I King Jordan

**Affiliations:** 1 School of Biology, Georgia Institute of Technology, Atlanta, GA 30332, USA; 2 National Center for Biotechnology Information, National Institutes of Health, 8600 Rockville Pike, Bethesda, MD 20894, USA

## Abstract

**Background:**

The majority of human non-protein-coding DNA is made up of repetitive sequences, mainly transposable elements (TEs). It is becoming increasingly apparent that many of these repetitive DNA sequence elements encode gene regulatory functions. This fact has important evolutionary implications, since repetitive DNA is the most dynamic part of the genome. We set out to assess the evolutionary rate and pattern of experimentally characterized human transcription factor binding sites (TFBS) that are derived from repetitive versus non-repetitive DNA to test whether repeat-derived TFBS are in fact rapidly evolving. We also evaluated the position-specific patterns of variation among TFBS to look for signs of functional constraint on TFBS derived from repetitive and non-repetitive DNA.

**Results:**

We found numerous experimentally characterized TFBS in the human genome, 7–10% of all mapped sites, which are derived from repetitive DNA sequences including simple sequence repeats (SSRs) and TEs. TE-derived TFBS sequences are far less conserved between species than TFBS derived from SSRs and non-repetitive DNA. Despite their rapid evolution, several lines of evidence indicate that TE-derived TFBS are functionally constrained. First of all, ancient TE families, such as MIR and L2, are enriched for TFBS relative to younger families like Alu and L1. Secondly, functionally important positions in TE-derived TFBS, specifically those residues thought to physically interact with their cognate protein binding factors (TF), are more evolutionarily conserved than adjacent TFBS positions. Finally, TE-derived TFBS show position-specific patterns of sequence variation that are highly distinct from random patterns and similar to the variation seen for non-repeat derived sequences of the same TFBS.

**Conclusion:**

The abundance of experimentally characterized human TFBS that are derived from repetitive DNA speaks to the substantial regulatory effects that this class of sequence has on the human genome. The unique evolutionary properties of repeat-derived TFBS are perhaps even more intriguing. TE-derived TFBS in particular, while clearly functionally constrained, evolve extremely rapidly relative to non-repeat derived sites. Such rapidly evolving TFBS are likely to confer species-specific regulatory phenotypes, *i.e*. divergent expression patterns, on the human evolutionary lineage. This result has practical implications with respect to the widespread use of evolutionary conservation as a surrogate for functionally relevant non-coding DNA. Most TE-derived TFBS would be missed using the kinds of sequence conservation-based screens, such as phylogenetic footprinting, that are used to help characterize non-coding DNA. Thus, the very TFBS that are most likely to yield human-specific characteristics will be neglected by the comparative genomic techniques that are currently *de rigeur *for the identification of novel regulatory sites.

## Background

The vast majority of the human genome is made up of non-protein-coding sequences [1,2], and the specific function of such DNA is often unknown. As of late, elucidating the functional relevance of the non-coding fraction of the human genome has become a major priority for computational and functional genomics [3].

Most of the non-protein-coding fraction of the human genome is made up of repetitive DNA sequences, primarily transposable elements (TEs), which alone make at least 45% of the genome. In one sense, these TEs can be considered as genomic parasites that exist solely by virtue of their ability to out-replicate the host genome in which they reside [4,5]. On the other hand, it has become abundantly clear that, once established in a genome, TEs can contribute to genome function in a number of different ways [6]. For instance, TEs are known to donate a wide variety of gene regulatory sequences to the human genome [7-9], and TE-derived regulatory sequences exert diversifying effects on the expression patterns of adjacent genes (reviewed in [10-12]).

TE-derived regulatory sequences are particularly interesting from an evolutionary perspective because of their potential to drive gene expression divergence between species. The potential for TEs to cause regulatory changes between evolutionary lineages is related to the fact that TEs invariably represent the most rapidly changing, lineage-specific part of eukaryotic genomes. For instance, when the human and mouse genomes sequences were compared, it became apparent that 99% of protein coding genes had human-mouse homologs, with 80% having direct 1:1 orthologs, whereas only 13% of mouse and 48% of human TEs were shared between the two species [13]. TE dynamics can even lead to substantial differences between genomes over relatively short evolutionary time scales. Indeed, the human evolutionary lineage has experience a TE-driven genome expansion of 500 Mb in the last 50 million years and 30 Mb since the divergence from chimpanzees [14].

Taken together with their ability to donate regulatory sequences, this lineage-specific character of TEs suggests that the regulatory elements they donate may lead to species-specific differences in gene expression. In fact, a primate-specific endogenous retroviral element has been shown to donate an enhancer that confers a distinct parotid-specific expression pattern on the human amylase gene [15]. A more recent genome scale analysis showed that TE-derived human regulatory sites are associated with genes that have increased tissue-specific expression divergence between human and mouse [16]. A corollary prediction of this model for the diversifying regulatory effects of TEs is that TE-derived regulatory sequences will have anomalously rapid evolutionary rates. Consistent with this expectation, we previously found that TE-derived human transcription factor binding sites (TFBS) are much less likely to have orthologs in the mouse genome than non-repetitive TFBS [17].

In this study, we set out to assess the relative evolutionary rates and the position-specific patterns of variation for human TFBS that are derived from repetitive versus non-repetitive DNA. We relied on the analysis of experimentally characterized TFBS that can be unambiguously mapped to the human genome in order to determine their evolutionary origins in repetitive or non-repetitive DNA. Our results suggest that TE-derived TFBS show both rapid evolution and, in some cases, anomalous position-specific patterns of change relative to non-repetitive TFBS. Despite these distinct evolutionary characteristics, the TE-derived TFBS do show sequence divergence patterns that are consistent with the conservation of function.

## Results and Discussion

### Human TFBS from repetitive DNA

A total of 2,521 experimentally characterized human TFBS were taken from the TRANSFAC database [18] and 1,810 of these were able to be precisely mapped to the latest build of the human genome reference sequence. Mapping of TFBS was done using the program site2genome, which facilitates unambiguous mapping of TFBS by using the longer flanking sequence context surrounding the relatively short binding sites [19]. The genomic locations of these human TFBS were compared to the locations of repetitive DNA sequences identified with the RepeatMasker program [20]. A total of 182 (10%) mapped human TFBS are co-located with repetitive DNA elements, and 121 (6.7%) of these are contained completely within repeats (Table 1). 62 of the TFBS derived completely from repeat regions are associated with TEs, while 59 are derived from simple sequence repeats (SSRs). SSRs are short tandem repeats consisting of repeated runs of exact or nearly exact *k*-mers, where *k *= 1–13 bp for microsattelites or *k *= 14–500 bp for minisatellites [1]. A lower percentage of the SSR co-located TFBS (57%) are found to completely overlap with the repeats compared to TE-derived TFBS (78%), suggesting that some of the SSR-derived TFBS identified here may represent ascertainment artifacts.

**Table 1:** Counts for human TFBS derived from repetitive DNA.

**Category**	**Total count**	**Complete overlap**	**Partial overlap**
All repeats	182	121	61
All SSR	103	59	44
All TEs	79	62	17
Alu	20	19	1
MIR	16	10	6
L1	10	4	6
All other LINEs	10	8	2
LTR	14	14	0
DNA	9	7	2

The numbers of experimentally characterized TFBS mapped to different categories of human genome sequence are shown. Total counts are indicated along with counts for those cases where the TFBS completely or partially overlaps with the repeat.

**Figure 1:**
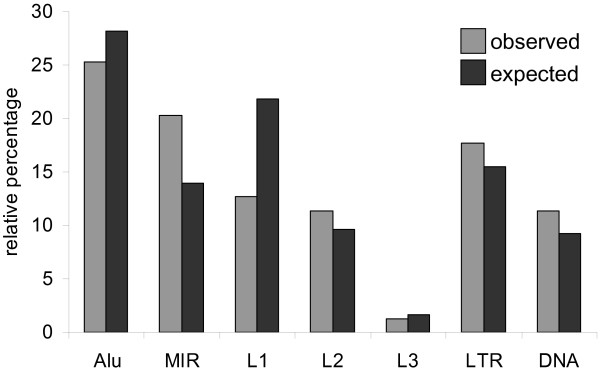
**Observed versus expected frequencies of TE-derived TFBS**. The observed percentages (light) of TE-derived TFBS from different classes/families of human TEs are plotted along with the percentages that are expected (dark) based on the background frequencies of the TEs in the genome. All class/family percentages are relative, *i.e*. they are normalized by the total number of TEs that donate TFBS (observed) and the total number of TEs in the genome (expected) respectively.

Human TEs can be characterized into specific classes/families, and the class/family-specific counts of TE-derived TFBS are shown in Table 1. The observed distributions of TE-derived TFBS across classes/families, relative to their expected distributions based on the genome frequencies of the TE classes/families, are shown in Figure 1. The human genome has experienced a number of successive waves of TE expansion, and accordingly, different TE families have distinct evolutionary ages [1]. For short interspersed nuclear elements (SINEs) and long interspersed nuclear elements (LINEs), relatively older families, such as MIR and L2, encode more TFBS than expected based on their genome frequencies, while proportionally fewer TFBS are derived from younger element families such as Alu and L1. The relative enrichment of TFBS encoded by older TE families is consistent with the action of purifying selection based on their regulatory function. In other words, these older elements are likely to have been preserved in the genome because of the regulatory sequences that they provide as was predicted by Silva *et al*. [21].

### Evolutionary sequence conservation of repeat-derived TFBS

**Figure 2:**
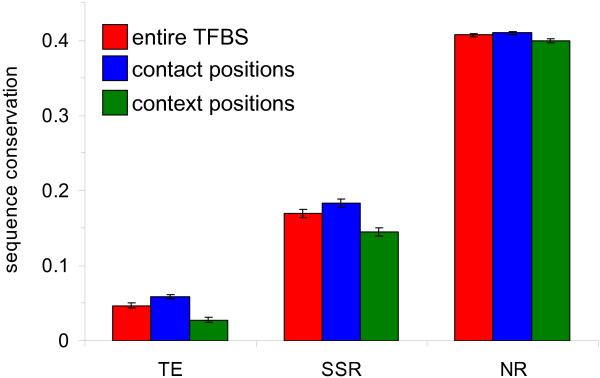
**Average evolutionary sequence conservation for repetitive versus non-repetitive TFBS**. Average conservation levels (± standard errors) are shown for TFBS that are derived from TEs, SSRs and non-repetitive DNA (NR). For each category, conservation levels were determined by averaging across the entire TFBS site (red), the specific contact part of the site that is thought to physically interact with the transcription factor (blue) and the sequence context part of the site that does contact the transcription factor (green).

Levels of evolutionary sequence conservation between 17 vertebrate species were compared for TFBS with origins in repetitive versus non-repetitive DNA (Figure 2). TE-derived TFBS are by far the least conserved of the three categories, followed by SSR-derived and then non-repetitive TFBS. All differences between these categories are highly statistically significant (110>*t*>19 0 = *P *< 9e-47). This pattern of low sequence conservation for the TE-derived TFBS is consistent with the prediction of our regulatory divergence model that TEs are prone to provide rapidly evolving, lineage-specific TFBS.

Having shown the high levels of sequence divergence for TE-derived TFBS, it is worth noting that evolutionary conservation is often taken as a measure of functional relevance. For instance, the phylogenetic footprinting approach identifies highly conserved regulatory sequences as more likely to be functional [22,23]. While a number of functionally relevant TE-derived sequences have recently been identified by virtue of their sequence conservation [24-28], the relatively unconserved TE-derived TFBS revealed by our analysis would almost certainly be overlooked by phylogenetic footprinting methods. However, the TFBS that we analyzed were experimentally characterized, not predicted, and are thus quite likely to represent *bona fide *functional regulatory elements. In fact, the analysis of the relative evolutionary rates for different positions in the TFBS described below demonstrates that the specific pattern of conservation across sites supports the assertion that the TE-derived TFBS are functional.

TRANSFAC annotations in the site table represent individual residues in TFBS with either upper-case or lower-case letters. The upper-case residues correspond to specific sequence motifs within the site that were emphasized by the authors of the cited literature. We consider upper-case residues to be more likely to form specific DNA-protein contacts. Accordingly, the upper- and lower-case TRANSFAC annotations were used to partition TFBS residues into putative 'contact' positions, which are thought to physically interact with transcription factors (TF), versus 'context' positions that make up the rest of the site. Presumably, putative contact positions are more functionally relevant than context positions, *i.e*. a change of sequence at a contact position would have more of an effect on TF binding than a change at a context position would. If this is indeed the case, then according to the phylogenetic footprinting rationale, contact positions should be more conserved than context positions. This prediction is confirmed for all three categories of TFBS seen in Figure 2, and all differences between conservation levels for contact versus context positions within categories are statistically significant (7.5>*t*>3.0 8.4e-11<*P *< 2.5e-3). In other words, although TE-derived TFBS do evolve more rapidly than the other categories of TFBS, the position-specific patterns of TE-TFBS sequence divergence are nonetheless consistent with selective constraint based on their regulatory function.

Evolutionary conservation rates for contact and context positions were further broken down for the different classes/families of TEs (Table 2). These data reveal several noteworthy trends. There are substantial differences in the level of conservation among classes and families. For instance, it is not surprising that the evolutionarily young Alu family of elements has the least conserved TFBS, and the young L1 family is similarly less conserved than the other older LINEs. One unexpected finding was the fact that TFBS derived from the long terminal repeats (LTRs) of endogenous retroviruses (ERVs) are the most conserved of all TE-derived TFBS. This observation stands out because ERVs are also evolutionarily young and not expected to be conserved. When this finding is considered together with the fact that LTRs are the only young class (or family) of TEs that has more TFBS than expected based on their genome frequencies (Figure 1), it suggest that LTRs may be particularly prone to donating regulatory sequences to the human genome. Indeed, LTRs are known to encode strong promoters, and there are a number of known cases where LTR-derived promoters control the expression of adjacent genes [29-33].

**Table 2:** Evolutionary sequence conservation of human TFBS.

**Category**	**Site**	**Contact**	**Context**
Non-repetitive	0.407 ± 0.085	0.410 ± 0.074	0.400 ± 0.110
All repeats	0.115 ± 0.042	0.130 ± 0.041	0.088 ± 0.045
All SSR	0.170 ± 0.056	0.183 ± 0.052	0.145 ± 0.062
All TEs	0.047 ± 0.026	0.059 ± 0.026	0.028 ± 0.026
Alu	0.002 ± 0.002	0.003 ± 0.003	0.002 ± 0.001
MIR	0.028 ± 0.017	0.048 ± 0.026	0.003 ± 0.004
L1	0.068 ± 0.063	0.077 ± 0.068	0.047 ± 0.052
All other LINEs	0.066 ± 0.018	0.095 ± 0.022	0.012 ± 0.011
LTR	0.141 ± 0.076	0.145 ± 0.042	0.136 ± 0.119
DNA	0.043 ± 0.029	0.057 ± 0.038	0.016 ± 0.009

Average (± standard deviation) base-by-base conservation levels are shown for different categories, non-repetitive and repetitive, of human TFBS. TFBS derived from repetitive DNA are broken down SSR versus TE-derived, and TE-derived TFBS are divided into specific classes/families of elements. Base-by-base conservation levels were averaged separately across entire sites, and across contact versus context positions.

Another relevant point from the class/family specific evolutionary conservation data is the fact that the relative rates of contact versus context TFBS position divergence are consistent across all categories observed (Table 2). The greater conservation of contact positions is seen for even the least conserved Alu family (*t *= 4.76 *P *= 2.7e-6). This indicates that the signal of functional constraint on TE-derived TFBS holds irrespective of the age of the elements from which the TFBS are derived, and serves as an independent confirmation of the experimental evidence in support of their identification.

### Position-specific variation patterns for TE-derived TFBS

**Figure 3:**
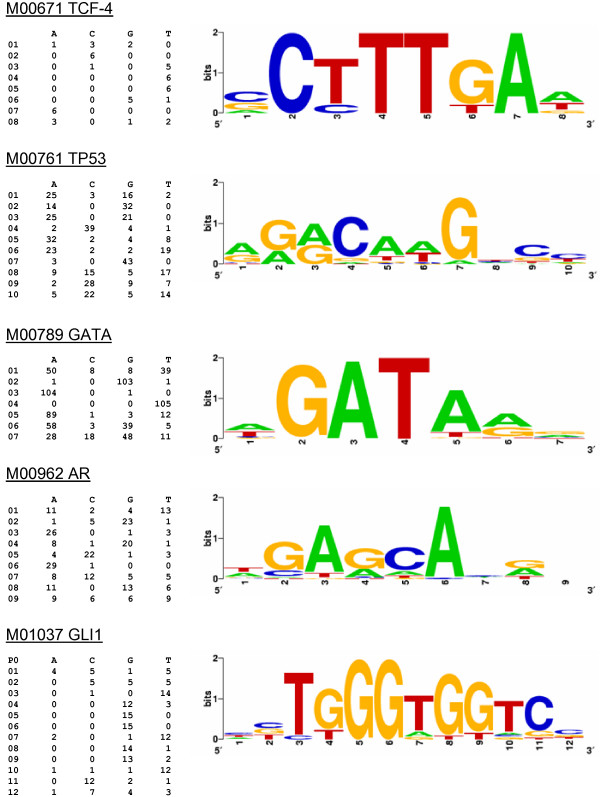
**Probabilistic modelling of TFBS**. PFMs for five collections of human TFBS (Table 3) are shown along with sequence logo representations of their PWMs. Each PFM/PWM represents a human TFBS that has both TE-derived and non-repetitive experimentally characterized sites in the genome. The TFBS are identified with their TRANSFAC matrix identifiers and the official human gene name symbol for the binding transcription factor proteins.

The results described in the previous section indicate that TE-derived TFBS show a low level of evolutionary conservation but a pattern of change that is consistent with their functional relevance as gene regulators. We used a probabilistic analysis of the position-specific patterns of sequence variation across TFBS sites to better understand the relative modes of evolution for non-repetitive versus TE-derived TFBS. To do this, position frequency matrices (PFMs) were taken from the TRANSFAC database for five TFBS where there was at least one TE-derived site in the human genome along with multiple non-repetitive TFBS. The PFMs summarize the collection of all experimentally characterized instances of that TFBS in the genome by representing the counts of each DNA residue (A, T, C or G) at each site in the TFBS (Figure 3). The PFMs can in turn be used to derive position weight matrices (PWMs), which are probabilistic representations of the position-specific nucleotide composition of the TFBS. The PWMs are represented as sequence logos [34], where the probabilities of observing a given residue at positions along the TFBS are indicated with the height of the residue symbols (Figure 3). We used these PWMs to score TE-derived versus non TE-derived TFBS sequences in terms of how well their specific sequences match the probabilistic model representing all other experimentally characterized sequences of that TFBS. The scoring was done using a 'leave-one-out' approach whereby each TFBS was scored using a PFM that does not include counts derived from the same TFBS. The TE-derived and non TE-derived sequence scores were compared to distributions of scores for three distinct simulated sets of 1,000 TFBS sequences. The first set of simulated TFBS sequences – 'genome-random' – was built by randomly drawing residues at each position of the TFBS based on their genome frequencies. The second set – 'repeat-random' – was generated from randomly sampled sequences, of the same length of the TFBS under consideration, taken from members of the same TE subfamily as the TE-derived TFBS being compared. Finally, the 'matrix-random' set was simulated according to the position-specific probabilities of the PWM for that TFBS.

**Figure 4:**
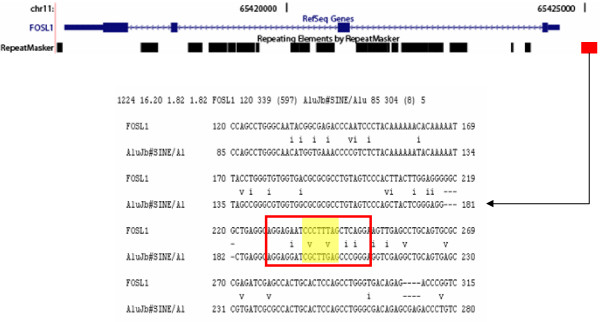
**An Alu-derived TFBS upstream of the FOSL1 encoding gene**. A schematic of the intron-exon structure of FOSL1, taken from the UCSC genome browser, is shown (blue) along with the positions of the repetitive DNA elements (black) at that locus. FOSL1 is encoded on the Crick strand of human chromosome 11. An Alu insertion (red) that donates a TCF-4 binding sites is found just upstream of the FOSL1 5' untranslated region in the proximal promoter region. Summary statistics and a sequence alignment between the FOSL1 proximal promoter sequence and the AluJb subfamily consensus sequence are shown with the TFBS location indicated (entire site boxed in red, contact residues highlighted in yellow).

An example of this kind of analysis can be seen for an Alu-derived TFBS (TRANSFAC site R08639) that sits just upstream of the FOS-like antigen (FOSL1)-encoding gene on human chromosome 11 (Figure 4). This TFBS was identified by virtue of its interaction with the beta-catenin-T cell-factor/lymphoid-enhancer-factor complex (Tcf/Lef) [35]. In that same study [35], binding of Tcf/Lef to FOSL1 and C-JUN was implicated in the progression of colon carcinoma. Interestingly, both FOSL1 and C-JUN are part of the AP-1 transcription complex suggesting that this Alu-derived TFBS may be involved in a cascade of regulatory interactions.

**Figure 5:**
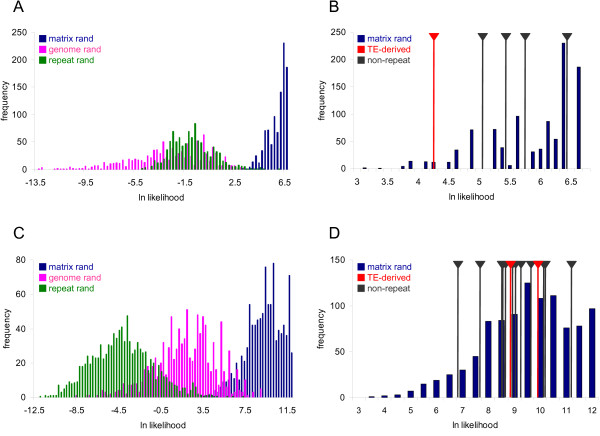
**Site-specific variation scores for TE-derived versus non-repetitive TFBS**. (A & C) Frequency distributions of scores for 1,000 simulated genome-random sequences (pink), repeat-random sequences (green) and matrix-random sequences (blue) for the M00671 matrix representing the TFBS bound by TCF-4 (A) and the M01037 matrix for TFBS bound by GLI1 (C). (B & D) The matrix-random score distributions are compared to the scores for individual TFBS derived from TEs (red) versus the non-repetitive TEs (gray). Data are shown for M00671 TCF-4 (B) and M01037 GLI1 (D).

The particular TRANSFAC PFM model that corresponds to this Alu-derived site is M00671, and the binding factor for this model is the T-cell-specific transcription factor 4 (TCF-4 aka TCF7L2). The PFM and derived PWM that correspond to the M00671 model are shown in Figure 3. This PWM was used to calculate scores for sets of genome-random, repeat-random and matrix-random sequences (Figure 5A). The Alu-derived and the non-repetitive TFBS were scored using PWMs built from M00671 PFMs that do not include residue counts from the particular TFBS being scored, *i.e*. using the leave-one-out method (Figure 5B). As could be expected, the genome-random and repeat-random simulated TFBS sequences have lower scores than do the matrix-random simulated sequences (Mann-Whitney U test *P *= 3.7e-5). What is more relevant is the fact that all of the experimentally characterized TFBS have scores that fall within the range of the matrix-simulated sequences and are much higher than either the genome-random or repeat-random scores (Table 3). This includes the Alu-derived TFBS, which scores significantly higher than the average scores for the genome-random and repeat-random sites (Mann-Whitney U test *P *= 1.9e-3). In other words, the Alu-derived TFBS has a position-specific DNA sequence profile that much more closely resembles the non TE-derived sites than it resembles random genomic sequences or random Alu sequences of the same subfamily. However, the Alu-derived site does have a lower score than all of the other non TE-derived sites. This indicates that there is still something unique about the TE-derived site relative to the non TE-derived sites. Thus, the position-specific profile of the Alu-derived TCF-4 binding site shows the hallmark of being functionally active yet retains a unique character relative to the non TE-derived sites that bind the same factor. The four other sites analyzed here show similar patterns in that they are clearly non-random, *i.e*. they score higher than the genome-random and repeat-random sets, and thus appear to be functional (Table 3). For the p53 matrix (M00761) Androgen receptor matrix (M00962), the TE-derived sites score lower than the non-repetitive sites; the two other cases show TE-derived sites with higher average scores than the non-repetitive sites. However, these differences are not statistically significant, indicating that TE-derived TFBS have position-specific profiles that are indistinguishable from non-repetitive TFBS. This is consistent with the fact that we started with experimentally characterized TFBS and underscores the functional relevance, and similar position-specific evolutionary constraints, of these TE-derived TFBS.

**Table 3:** Position-specific sequence variation scores for TE-derived, non-repetitive, matrix-random and genome-random TFBS.

**Matrix^1^**	**Protein binding factor^2^**	**TE-derived**	**non-repetitive**	**matrix-rand**	**genome-rand**	**repeat-rand**
M00671	T-cell-specific transcription factor 4 (TCF-4 or TCF7L2)	4.25	5.69 ± 0.51	5.80 ± 0.73	-48.76 ± 15.61	-48.63 ± 14.97
M00761	p53 (TP53)	5.97	6.65 ± 1.26	5.52 ± 1.92	-2.79 ± 3.02	-4.71 ± 3.35
M00789	GATA binding proteins (GATA)	6.12	5.26 ± 1.56	5.27 ± 1.46	-5.87 ± 2.71	-4.70 ± 3.15
M00962	Androgen receptor (AR)	3.72	4.45 ± 1.21	4.33 ± 1.74	-2.29 ± 1.28	-1.80 ± 2.17
M01037	Glioma-associated oncogene homolog 1 (GLI1)	9.34	9.12 ± 1.14	9.24 ± 1.70	1.77 ± 2.83	-4.28 ± 2.91

Average TFBS scores are shown for each category of sites.

^1^The TRANSFAC database matrix identifier

^2^The colloquial name of the protein that binds the TFBS along with its official HUGO name in parentheses

## Conclusion

There are numerous experimentally characterized TFBS in the human genome (7–10%) that are derived from repetitive DNA indicating a pronounced effect of repetitive DNA on human gene regulation. TFBS that originate from repeats evolve more rapidly than non-repetitive TFBS but still shown signs of sequence conservation on functionally critical residues due to purifying selection. Position-specific patterns sequence variation observed for TE-derived TFBS, in terms of the specific nucleotide composition along the positions of the TFBS, also point to divergence in the face of functional constraint. These findings are consistent with the notion that TFBS originating from repetitive DNA elements are likely to provide functionally relevant regulatory divergence between species.

## Methods

Experimentally characterized human transcription factor binding sites (TFBS) were retrieved from the Professional release 11.3 (9/10/07) of the TRANSFAC database [18]. These TFBS were mapped to the July 2003 human reference sequence [1] (National Center for Biotechnology (NCBI) Build 34 or hg16) using the program site2genome [19]. For many individual TFBS, TRANSFAC annotations list GenBank accessions that provide longer flanking sequence context for the relatively short TFBS contained within the sequence. Site2genome uses this flanking sequence context to allow for one-to-one TFBS-to-genome mapping. Only TFBS that could be unambiguously mapped to the human genome sequence (1,810 out of 2,521) were taken for further analysis, and these TFBS mappings were transferred to the current human genome build (NCBI Build 36 or hg18) using the UCSC Genome Browser [36] 'liftover' utility. The locations of human TFBS were compared to the locations of repetitive DNA, transposable elements (TEs) and simple sequence repeats (SSRs), annotated with the RepeatMasker program [20].

The evolutionary conservation levels for human TFBS were determined based on complete genome sequence alignments [37] between the human genome and 16 other vertebrate genomes [38]. These alignments have been analyzed, along with the phylogenetic tree of the species, by the program phastCons [39] to make predictions of discrete conserved genomic elements and to produce conservation level scores for each position (base) in the human genome. The base-by-base conservation level scores range from 0 to 1 and represent the posterior probability of every individual position in the genome being in a conserved element. Base-by-base conservation level scores were taken across all positions of the mapped TFBS and then averaged for the different categories compared in Table 2 and Figure 2.

Individual TFBS were broken down into putative contact and context positions using the TRANSFAC site table annotations. In the site table, the TFBS sequences are represented with upper-case and lower-case residues. The upper-case TFBS residues correspond to specific sequence motifs within the site that were emphasized by the authors of the cited literature. We consider upper-case residues to be more likely to form specific DNA-protein contacts than lower case residues. Accordingly, the upper- and lower-case TRANSFAC annotations were used to partition TFBS residues into putative 'contact' positions, which are thought to physically interact with transcription factors (TF), versus 'context' positions. TFBS were also divided into those derived from repetitive, TE and SSR, versus non-repetitive classes and average conservation scores were determined for each TFBS class over each residue (contact and context) class. The statistical significance of the differences between average evolutionary conservation levels was evaluated using the Students' t-test.

Analysis of the site-specific pattern of TFBS evolution was done using probabilistic models of TFBS that were computed based on a previously described protocol [40]. Position frequency matrices (PFMs), which represent the counts of each of the four DNA residues (A, T, C and G) in each position of a TFBS model, were downloaded from TRANSFAC 10.3. PFMs were converted into position-weight matrices (PWMs), which represent the probability (*p*) of observing each DNA residue (*r*) at each position (*i*) in a TFBS according to the following formula:

$$p_{r,i}=\frac{c_{r,i}+s_{r}}{n+4*s_{r}}$$

where *c*_*r*, *i *_= counts of residue *r *at position *i*, *s*_*r *_is a pseudocount function = 1, and *n *= the total number of TFBS used to build the model. These probabilities (*p*_*r*, *i*_) are normalized by the background genome frequencies of the DNA residues (*p*_*r*_) to compute weights (*W*):

*W*_*r*, *i *_= *p*_*r*, *i*_/*p*_*r*_

The PWMs are represented as sequence logos [34], which were built from the collections of TFBS sequences provided by the TRANSFAC matrix database, using the program WebLogo [41]. PWMs were used in Monte-Carlo simulation to build test sets of 1,000 TFBS sequences, the so-called 'matrix-random' sequences. For this procedure, DNA residues at each position of a TFBS were drawn at random according the site-specific probabilities of its PWM. 'Genome-random' simulated sets of 1,000 TFBS were built by randomly drawing residues across site positions according to their background genome frequencies. 'Repeat-random' simulated sets of 1,000 TFBS were generated by randomly sampling sequences of the same length of the matrix from members of the same repeat (TE) subfamily that the particular TE-derived TFBS was derived. The PWMs were used compute scores (*S*) individual observed and simulated TFBS according to the formula:

$$S=\sum_{i=1}^{n}\ln W_{r,i}$$

where *W*_*r*, *i *_= the weight of the observed residue *r *at position *i *and *n *= the number of sites in the TFBS PWM. Individual TFBS from the TRANSFAC site table were scored using the leave-one-out method whereby matrix-specific PFMs were iteratively built without residue counts from the particular TFBS being scored. Scores (*S*) were compared for individual TE-derived and non-repetitive TFBS along with the score distributions for simulated sets of matrix-random and genome-random sites.

## Authors' contributions

IKJ and LM–R conceived of and designed the study and performed computational analyses. LM–R and DL provided data used for the computational analyses. NP performed computational analyses in the lab of JFMcD. IKJ drafted the manuscript. All authors read and approved of the manuscript.

## References

[B1] Lander ES, Linton LM, Birren B, Nusbaum C, Zody MC, Baldwin J, Devon K, Dewar K, Doyle M, FitzHugh W (2001). Initial sequencing and analysis of the human genome. Nature.

[B2] Venter JC, Adams MD, Myers EW, Li PW, Mural RJ, Sutton GG, Smith HO, Yandell M, Evans CA, Holt RA (2001). The sequence of the human genome. Science.

[B3] Consortium EP (2004). The ENCODE (ENCyclopedia Of DNA Elements) Project. Science.

[B4] Doolittle WF, Sapienza C (1980). Selfish genes, the phenotype paradigm and genome evolution. Nature.

[B5] Orgel LE, Crick FH (1980). Selfish DNA: the ultimate parasite. Nature.

[B6] Kidwell MG, Lisch DR (2001). Perspective: transposable elements, parasitic DNA, and genome evolution. Evolution Int J Org Evolution.

[B7] Jordan IK, Rogozin IB, Glazko GV, Koonin EV (2003). Origin of a substantial fraction of human regulatory sequences from transposable elements. Trends Genet.

[B8] Thornburg BG, Gotea V, Makalowski W (2006). Transposable elements as a significant source of transcription regulating signals. Gene.

[B9] Lagemaat LN van de, Landry JR, Mager DL, Medstrand P (2003). Transposable elements in mammals promote regulatory variation and diversification of genes with specialized functions. Trends Genet.

[B10] Britten RJ (1996). DNA sequence insertion and evolutionary variation in gene regulation. Proc Natl Acad Sci USA.

[B11] Britten RJ (1997). Mobile elements inserted in the distant past have taken on important functions. Gene.

[B12] Medstrand P, Lagemaat LN van de, Dunn CA, Landry JR, Svenback D, Mager DL (2005). Impact of transposable elements on the evolution of mammalian gene regulation. Cytogenet Genome Res.

[B13] Waterston RH, Lindblad-Toh K, Birney E, Rogers J, Abril JF, Agarwal P, Agarwala R, Ainscough R, Alexandersson M, An P (2002). Initial sequencing and comparative analysis of the mouse genome. Nature.

[B14] Liu G, Zhao S, Bailey JA, Sahinalp SC, Alkan C, Tuzun E, Green ED, Eichler EE (2003). Analysis of primate genomic variation reveals a repeat-driven expansion of the human genome. Genome research.

[B15] Samuelson LC, Wiebauer K, Snow CM, Meisler MH (1990). Retroviral and pseudogene insertion sites reveal the lineage of human salivary and pancreatic amylase genes from a single gene during primate evolution. Mol Cell Biol.

[B16] Marino-Ramirez L, Jordan IK (2006). Transposable element derived DNaseI-hypersensitive sites in the human genome. Biol Direct.

[B17] Marino-Ramirez L, Lewis KC, Landsman D, Jordan IK (2005). Transposable elements donate lineage-specific regulatory sequences to host genomes. Cytogenet Genome Res.

[B18] Matys V, Fricke E, Geffers R, Gossling E, Haubrock M, Hehl R, Hornischer K, Karas D, Kel AE, Kel-Margoulis OV (2003). TRANSFAC: transcriptional regulation, from patterns to profiles. Nucleic Acids Res.

[B19] Frith MC, Halees AS, Hansen U, Weng Z (2004). Site2genome: locating short DNA sequences in whole genomes. Bioinformatics.

[B20] RepeatMasker. http://www.repeatmasker.org/.

[B21] Silva JC, Shabalina SA, Harris DG, Spouge JL, Kondrashovi AS (2003). Conserved fragments of transposable elements in intergenic regions: evidence for widespread recruitment of MIR- and L2-derived sequences within the mouse and human genomes. Genet Res.

[B22] Gumucio DL, Heilstedt-Williamson H, Gray TA, Tarle SA, Shelton DA, Tagle DA, Slightom JL, Goodman M, Collins FS (1992). Phylogenetic footprinting reveals a nuclear protein which binds to silencer sequences in the human gamma and epsilon globin genes. Mol Cell Biol.

[B23] Zhang Z, Gerstein M (2003). Of mice and men: phylogenetic footprinting aids the discovery of regulatory elements. J Biol.

[B24] Bejerano G, Lowe CB, Ahituv N, King B, Siepel A, Salama SR, Rubin EM, Kent WJ, Haussler D (2006). A distal enhancer and an ultraconserved exon are derived from a novel retroposon. Nature.

[B25] Kamal M, Xie X, Lander ES (2006). A large family of ancient repeat elements in the human genome is under strong selection. Proc Natl Acad Sci USA.

[B26] Lowe CB, Bejerano G, Haussler D (2007). Thousands of human mobile element fragments undergo strong purifying selection near developmental genes. Proc Natl Acad Sci USA.

[B27] Nishihara H, Smit AF, Okada N (2006). Functional noncoding sequences derived from SINEs in the mammalian genome. Genome research.

[B28] Xie X, Kamal M, Lander ES (2006). A family of conserved noncoding elements derived from an ancient transposable element. Proc Natl Acad Sci USA.

[B29] Bannert N, Kurth R (2004). Retroelements and the human genome: new perspectives on an old relation. Proc Natl Acad Sci USA.

[B30] Dunn CA, Medstrand P, Mager DL (2003). An endogenous retroviral long terminal repeat is the dominant promoter for human beta1,3-galactosyltransferase 5 in the colon. Proc Natl Acad Sci USA.

[B31] Dunn CA, Romanish MT, Gutierrez LE, Lagemaat LN van de, Mager DL (2006). Transcription of two human genes from a bidirectional endogenous retrovirus promoter. Gene.

[B32] Romanish MT, Lock WM, Lagemaat LN van de, Dunn CA, Mager DL (2007). Repeated recruitment of LTR retrotransposons as promoters by the anti-apoptotic locus NAIP during mammalian evolution. PLoS Genet.

[B33] Wang T, Zeng J, Lowe CB, Sellers RG, Salama SR, Yang M, Burgess SM, Brachmann RK, Haussler D (2007). Species-specific endogenous retroviruses shape the transcriptional network of the human tumor suppressor protein p53. Proc Natl Acad Sci USA.

[B34] Schneider TD, Stephens RM (1990). Sequence logos: a new way to display consensus sequences. Nucleic Acids Res.

[B35] Mann B, Gelos M, Siedow A, Hanski ML, Gratchev A, Ilyas M, Bodmer WF, Moyer MP, Riecken EO, Buhr HJ (1999). Target genes of beta-catenin-T cell-factor/lymphoid-enhancer-factor signaling in human colorectal carcinomas. Proc Natl Acad Sci USA.

[B36] Kent WJ, Sugnet CW, Furey TS, Roskin KM, Pringle TH, Zahler AM, Haussler D (2002). The human genome browser at UCSC. Genome research.

[B37] Blanchette M, Kent WJ, Riemer C, Elnitski L, Smit AF, Roskin KM, Baertsch R, Rosenbloom K, Clawson H, Green ED (2004). Aligning multiple genomic sequences with the threaded blockset aligner. Genome research.

[B38] Vertebrate Multiz Alignment & Conservation (17 Species). http://www.genome.ucsc.edu/cgi-bin/hgTrackUi?hgsid=100603286&c=chrX&g=multiz17way.

[B39] Siepel A, Bejerano G, Pedersen JS, Hinrichs AS, Hou M, Rosenbloom K, Clawson H, Spieth J, Hillier LW, Richards S (2005). Evolutionarily conserved elements in vertebrate, insect, worm, and yeast genomes. Genome research.

[B40] Wasserman WW, Sandelin A (2004). Applied bioinformatics for the identification of regulatory elements. Nat Rev Genet.

[B41] Crooks GE, Hon G, Chandonia JM, Brenner SE (2004). WebLogo: a sequence logo generator. Genome research.

